# Case Report: A rare case of renal failure secondary to in utero megalourethra

**DOI:** 10.3389/fped.2025.1546561

**Published:** 2025-05-08

**Authors:** S. Ali, V. Nunez, R. Terkawi, C. Katsoufis, C. Abitbol, R. Ruano, J. Duara, T. Fontanez-Nieves

**Affiliations:** ^1^ Division of Neonatology, University of Miami Miller School of Medicine, and Holtz Children’s Hospital, Miami, FL, United States; ^2^ Division of Pediatric Nephrology, University of Miami Miller School of Medicine, and Holtz Children’s Hospital, Miami, FL, United States; ^3^ Division of Maternal Fetal Medicine/Fetal Surgery, University of Miami Miller School of Medicine, and Holtz Children’s Hospital, Miami, FL, United States

**Keywords:** congenital megalourethra, anhydramnios, renal failure, pulmonary hypoplasia, bladder

## Abstract

**Background:**

Megalourethra is a rare congenital condition marked by dilation and elongation of the penile urethra, resulting from hypoplasia of either the corpus spongiosum or corpus cavernosa.

**Case presentation:**

We describe a novel case of a male infant prenatally diagnosed with megalourethra who subsequently developed rapid-onset anhydramnios and advanced renal failure.

**Conclusion:**

Unlike previously described cases, this case is unique due to the patient's abrupt progression to anhydramnios at 34 weeks and 5 days, despite having normal amniotic fluid levels prior to that. With late-onset anhydramnios, the severity of renal dysfunction was unexpected. Proximal urethrostomy requires further assessment as a potential intervention to successfully bypass the megalourethra and prevent infection. Long-term management is expected to include dialysis as a bridge to transplantation. Multiple corrective urological surgeries will be required to repair the urethra and restore penile function. Timely prenatal diagnosis of megalourethra or renal anomalies is essential for predicting long-term prognosis, as these conditions can lead to significant postnatal complications.

## Introduction

Megalourethra is a rare urogenital malformation characterized by the dilation and elongation of the penile urethra, often associated with the absence or hypoplasia of the corpus spongiosum and cavernosa. It is usually diagnosed prenatally through ultrasound and can lead to significant postnatal complications, including voiding and erectile dysfunction, renal insufficiency, and pulmonary hypoplasia. To date, fewer than 100 cases have been documented in the literature (1). There are two types of congenital megalourethra: scaphoid, the most common type, characterized by deficiency or complete absence of the corpus spongiosum, and fusiform, which involves malformation of both the corpora cavernosa and corpus spongiosum. The fusiform type is more severe and often associated with other congenital malformations (2). Here, we present a case of a male infant diagnosed prenatally with megalourethra, who unexpectedly developed anhydramnios 12 weeks after diagnosis. This is rare, as previous case reports have shown that patients develop anhydramnios relatively sooner after diagnosis (3). Moreover, our patient was born with severe kidney failure, which was unpredictable (4).

## Case presentation

**Figure 1:**
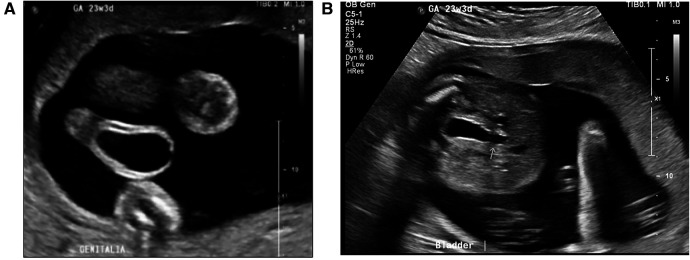
**(A)** Antenatal ultrasound at 23 + 3 weeks gestation, showing a ventral view of dilated megalourethra. **(B)** Presence of keyhole sign on the antenatal ultrasound.

A male infant was born to a 28-year-old primigravida at a gestational age of 35 weeks and 1 day. The pregnancy was complicated by gestational diabetes and preeclampsia without severe features. An antenatal ultrasound at 22 weeks revealed a fetus with megalourethra associated with mild bilateral hydroureters and mildly dilated bladder but a normal amount of amniotic fluid. Follow-up ultrasound at 23 weeks and 3 days showed normal renal echogenicity and presence of a keyhole sign, which was not mentioned on the previous ultrasound, and mild thickening of the bladder wall was noted (Figures 1A,B).

Follow-up ultrasound examinations showed stable findings until 34 weeks and 5 days, when an abrupt decrease in the amount of amniotic fluid was observed, with a maximum vertical pocket measuring <1 cm. Preterm premature rupture of membranes was ruled out by a negative pH test. Strict pad counts also confirmed no leakage of fluid. There was no evidence of rupture or fluid leak either subjectively or through medical testing.

**Figure 2:**
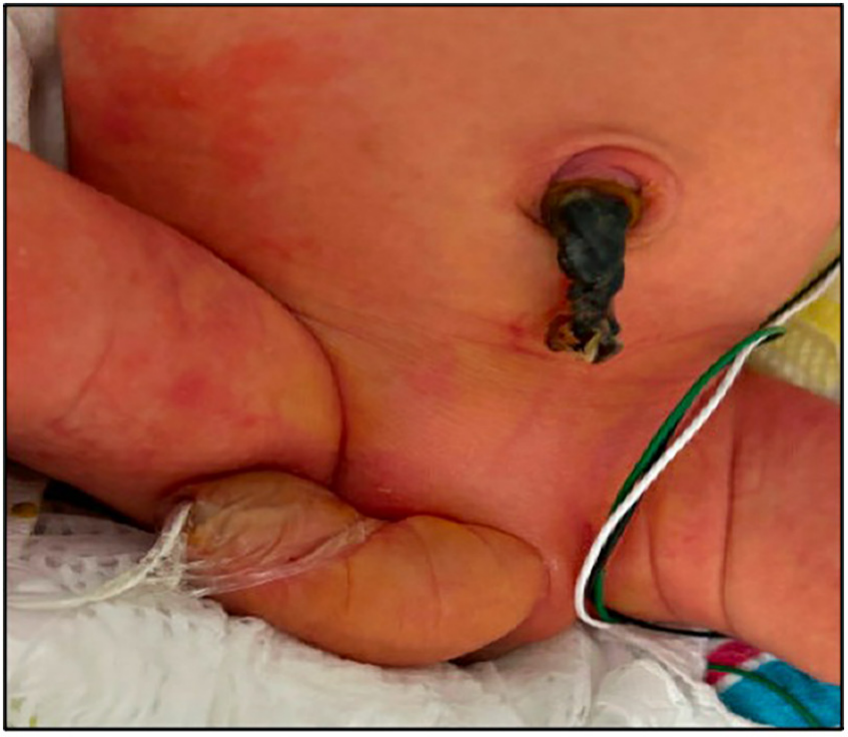
Postnatal photograph in the first week of life, showing macrophallus with dorsal curvature, scaphoid-type megalourethra.

Prior to delivery, the parents were counseled regarding postnatal outcomes, the need for possible surgical intervention, and potential renal impairment. Given the abrupt onset of anhydramnios and the fact that the patient was not very premature, a risk-versus-benefit decision was made to deliver the baby early via an urgent cesarean section. A male infant weighing 2.56 kg was delivered which corresponds to the 63rd percentile per the Fenton 2013 Growth Calculator. Clinical examination indicated a curved penis with length measuring 6–8 cm in length, along with excess skin (Figure 2).

**Figure 3:**
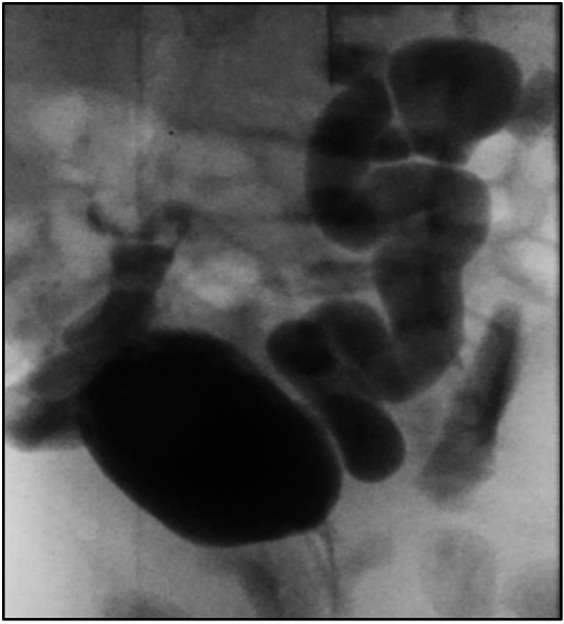
Voiding cystourethrogram (VCUG) indicated bilateral grade 5 vesicoureteral reflux.

After delivery, the infant developed spontaneous small bilateral pneumothoraxes, which resolved within a few days without acute intervention. Severe renal anomalies have been associated with spontaneous pneumothoraxes; however, in our patient, this was unexpected as there was no sonographic evidence of significant renal failure prenatally (5). He did not require oxygen supplementation or respiratory support. The baby voided immediately after birth. A postnatal ultrasound showed bilateral renal dysplasia with bilateral dilated ureters, and a voiding cystourethrogram (VCUG) indicated bilateral grade 5 vesicoureteral reflux without posterior urethral valves (Figure 3). Whole-genome sequencing did not identify any abnormalities.

The infant progressed to severe, non-oliguric renal insufficiency, managed conservatively with a low salt formula and a potassium binder. The peak serum creatinine was 5.9 mg/dl, stabilizing between 3 and 4 mg/dl before discharge. Despite receiving prophylactic antibiotics, he experienced three urinary tract infections (UTIs) within the first 3 months of life. After the first two infections, a perineal urethrostomy was performed to minimize urine pooling. Although vesicostomy is often considered a preferable diversion option, its invasiveness and associated risks led to the decision to pursue urethrostomy, which may prevent infections through a less invasive procedure. However, 2 weeks after discharge from the neonatal intensive care unit, he developed another episode of febrile urosepsis. The patient's family relocated, and he was started on peritoneal dialysis at around four months of life.

## Discussion

Megalourethra can present as an isolated cosmetic disfigurement or as part of syndromic associations such as the VACTERL association, Eagle–Barrett syndrome, and Potter's sequence (6). Our patient had the “scaphoid” type with severe malformation of the corpora cavernosa and corpus spongiosum. He did not meet the criteria for any congenital syndromes or associations.

Contrary to a report detailing four pregnancies with megalourethra (3), our patient experienced late-onset anhydramnios without any evidence of pulmonary hypoplasia. In that report, three of the infants died shortly after birth due to pulmonary hypoplasia, and one case resulted in termination. Among the three infants, one was diagnosed with oligohydramnios at 28 weeks of gestation and delivered via cesarean section. Another was diagnosed at 32 weeks and delivered vaginally, while the third had severe oligohydramnios diagnosed earlier in the pregnancy. Our patient had normal amniotic fluid at 33 weeks but developed severe oligohydramnios about 11 days later. He did not develop pulmonary hypoplasia, possibly dueto the brief duration of oligohydramnios and its late onset (7).

The patient also presented with significant renal dysplasia and congenital renal failure. A larger retrospective study of 50 cases observed that early-onset oligohydramnios was more strongly associated with impaired kidney function. The average onset of oligohydramnios in patients with renal impairment was 17.8 ± 3.6 weeks, compared to 29.5 ± 9.2 weeks in those with normal renal function (*p* = 0.03) (4). Among these 50 patients, 52% were noted to have megacystis, 58% patients with bilateral hydronephrosis, and 48% patients with bilateral hydroureters. Renal function was reported in 31 patients and was normal in 58% (4). The principle of non-anatomical functional obstruction of the urethra caused by balloon-like pressure has been described (7, 8). Our patient had mild ureteral dilation and normal amniotic fluid until late pregnancy, making significant renal impairment unexpected. Moreover, his course was further complicated by recurrent UTIs, which are common in congenital megalourethra (9).

Surgical management of congenital megalourethra varies based on the type. For scaphoid megalourethra, a one- or two-step urethroplasty is commonly performed to reduce urethral size and improve cosmetic outcomes. For the fusiform type, treatment options include urethroplasty or, in some cases, sex reassignment surgery (6, 10). In our patient's case, severe vesicoureteral reflux (VUR) and ureteral involvement warranted a temporizing urethrostomy to prevent further renal failure progression. Similar cases in the literature, such as one described by Hata et al., reported patients with VUR and renal failure who required urethrostomy and eventually progressed to advanced renal disease (11).

## Conclusion

This case highlights the rapid progression to anhydramnios and significant renal dysfunction, emphasizing the unique presentation and variability of megalourethra. Following discharge from our neonatal intensive care unit, the patient was transferred to an outside hospital, where peritoneal dialysis was initiated as a bridge to kidney transplantation. Given the rarity of reported cases, further research is needed to improve our understanding of this condition. Long-term management will involve dialysis until transplantation, along with multiple corrective urological surgeries to restore urethral and penile function. Early prenatal diagnosis of megalourethra or renal anomalies is crucial for anticipating long-term outcomes, as these conditions can result in severe postnatal complications. A thorough prenatal evaluation and a multidisciplinary approach are essential for optimizing care and improving patient outcomes.

## Data Availability

The raw data supporting the conclusions of this article will be made available by the authors, without undue reservation.
